# The human microbiome and cancer: a diagnostic and therapeutic perspective

**DOI:** 10.1080/15384047.2023.2240084

**Published:** 2023-07-27

**Authors:** Shruthi Kandalai, Huapeng Li, Nan Zhang, Haidong Peng, Qingfei Zheng

**Affiliations:** a Department of Radiation Oncology, College of Medicine, The Ohio State University, Columbus, OH, USA; b Center for Cancer Metabolism, James Comprehensive Cancer Center, The Ohio State University, Columbus, OH, USA; c Molecular, Cellular, and Developmental Biology Graduate Program, The Ohio State University, Columbus, OH, USA; d Department of Biological Chemistry and Pharmacology, College of Medicine, The Ohio State University, Columbus, OH, USA

**Keywords:** human microbiome, cancer diagnostics, cancer development, cancer therapy, immune response

## Abstract

Recent evidence has shown that the human microbiome is associated with various diseases, including cancer. The salivary microbiome, fecal microbiome, and circulating microbial DNA in blood plasma have all been used experimentally as diagnostic biomarkers for many types of cancer. The microbiomes present within local tissue, other regions, and tumors themselves have been shown to promote and restrict the development and progression of cancer, most often by affecting cancer cells or the host immune system. These microbes have also been shown to impact the efficacy of various cancer therapies, including radiation, chemotherapy, and immunotherapy. Here, we review the research advances focused on how microbes impact these different facets and why they are important to the clinical care of cancer. It is only by better understanding the roles these microbes play in the diagnosis, development, progression, and treatment of cancer, that we will be able to catch and treat cancer early.

## Introduction

The human microbiome contains a diverse set of bacteria, viruses, fungi, protozoa, and archaea that exist on and within the human body. It has been estimated that the human-associated bacteria alone outnumber human cells in the body.^1^ While the majority of these microbes may not directly harm humans, they can have significant effects on human health. Though it has been estimated that there are trillions of organisms in the human microbiome, the International Agency for Research on Cancer (IARC) currently designates only 11 as being directly carcinogenic (Group 1 carcinogens) to humans.^2^ These 11 organisms include one species of bacteria (*Heliobacter pylori)*, seven species of viruses (Epstein-Barr virus, hepatitis B virus, hepatitis C virus, Kaposi sarcoma virus, human immunodeficiency virus-1, human papillomaviruses, and human T-cell lymphotropic virus), and three species of parasitic worms (*Opisthorchis viverrini*, *Clonorchis sinensis*, and *Schistosoma haematobium*) and together, are responsible for about 2.2 million cancer cases annually worldwide.^2^ The IARC has further categorized other microbes, such as protozoan parasite *Plasmodium falciparum* and multiple polyomaviruses, as being probably carcinogenic (Group 2A) or possibly carcinogenic (Group 2B)^3^, as the current data is insufficient to show that these are directly carcinogenic. The IARC is also currently evaluating additional microbes for their carcinogenicity, including bacterium *Salmonella typhi* and its association with gallbladder cancer, human cytomegalovirus with multiple cancer types, parasitic worm *Schistosoma mansoni* with multiple cancer types, hepatitis D virus with liver cancer, and parasitic worm *Opisthorchis felineus* with bile duct cancer.^4^

**Figure 1.**
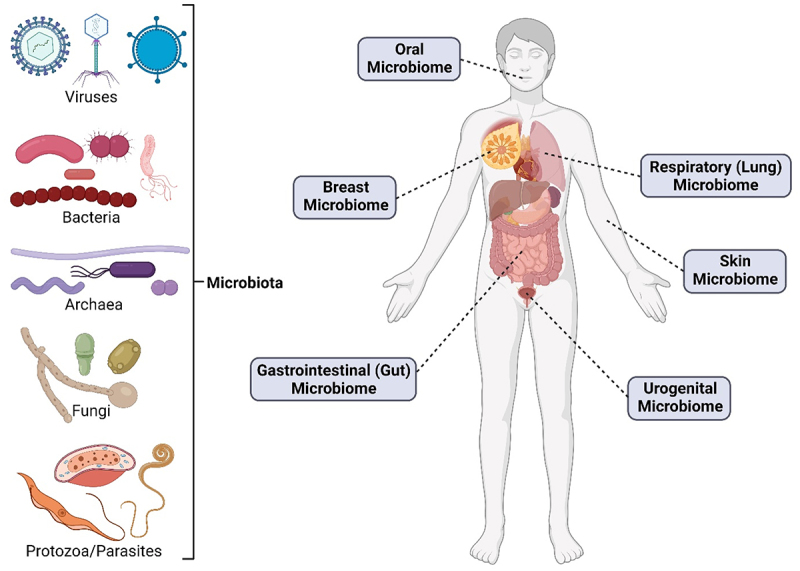
Various types of microbes (left) that have been found to make up different human microbiomes (right).

While the IARC mostly focuses on categorizing agents that directly cause cancer, many more have been shown to indirectly cause cancer. It is for this reason that studying the normal microbial flora (Figure 1) of different regions and how dysbiosis may occur is imperative to better understanding how microbes affect cancer. This is also reflected in the recent addition of polymorphic microbes as an enabling characteristic in the hallmarks of cancer.^5^ One of the most heavily studied regions of the human microbiota is that of the gut. The gut microbiome was a focus of the Human Microbiome Project and in other studies that followed, and has been implicated in cancer susceptibility and carcinogenesis for over a decade, though many of the causal relationships between these gut microbes and disease are still being understood.^6^ One of the most notable and researched examples of a gut microbe implicated in cancer development is colibactin-producing *Escherichia coli*. Colibactin has been found to trigger DNA double-stranded breaks,^7^ cause chromosomal instability,^8^ and alkylate DNA,^9^ and for these reasons, has been found to be involved in colorectal cancer (CRC) development.^10^ More recently, there has been an increasing interest in the microbiome of regions previously thought to be sterile and how changes to these regions may promote local cancer development and progression,^11–13^ Following recent advances in characterization methods^14^, there has also been an emerging focus on the tumor microbiome, or organisms that are present within the tumor microenvironment or the tumor cells themselves, and how these microbes may serve to improve the fitness and immune evasion of cancer cells,^15^

In this review, we highlight how the microbiome can directly and indirectly affect many aspects of cancer care, from disease development and progression to diagnostics and therapeutics. We discuss how recent literature has changed the narrative on how these interactions could impact cancer care. Finally, we outline why understanding these previously elusive interactions between microbes and cancer will serve to better cancer diagnoses and treatments in the future.

## The influence of microbiome on cancer development and progression

**Figure 2.**
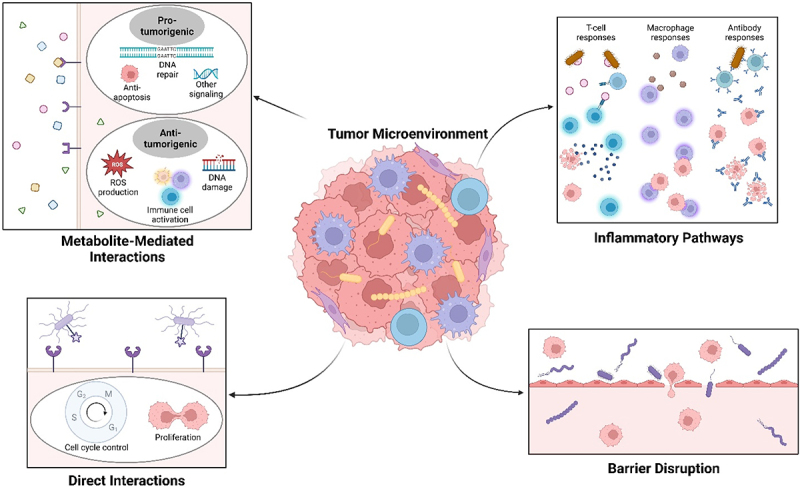
As microbes are present in local tissues, the tumor microenvironment (middle), and within tumor cells themselves, these tumor-associated microbes have been found to impact different cancer types through different methods, including metabolite-mediated interactions that can be pro-tumorigenic or anti-tumorigenic (top left); direct interactions with cancer cells to control the cell cycle and proliferation (bottom left); activation of inflammatory cells (glowing cells), such as T-cells (in blue), macrophages (in purple), and antibodies (in blue) (top right); and by disrupting vascular barriers to promote metastasis (bottom right).

Microbes have been implicated in indirectly affecting many types of cancer (Figure 2). For example, gut microbes have been shown to impact cancer stem cells, or cancer cells that become quiescent and are thought to be responsible for disease relapse.^16^ We have also previously shown that metabolites from archaea, specifically the phyla *Euryarchaeota* and the TACK superphylum (*Thaumarchaeota*, *Aigarchaeota*, *Crenarchaeota*, and *Korarchaeota*), are associated with many types of cancer, despite these microbes often being disregarded due to their low prevalence.^17^ By and large, the majority of research linking microbes and cancer has been focused on bacteria and their roles in a variety of cancers, though some have also covered the possible associations of fungi and viruses.

### Colorectal cancer

As mentioned previously, the first gut metabolite implicated in cancer development was colibactin, which is produced by *E. coli* and other members of the family *Enterobacteriaceae* and has been found to be involved in CRC development.^7–10^ Since the discovery of colibactin, there has been additional interest in how other gut microbes, including those thought to be commensals like *E. coli*, may affect CRC development and progression. Bacterial pathogen *Helicobacter hepaticus* has also been shown to significantly increase the incidence of colon tumors in an inflammatory-based mouse model of colorectal cancer, either due to increased inflammation^18^ or the production of virulence factors by this species.^19^ Species belonging to genera *Bacteroides* and *Prevotella* have also been noted as being significantly more abundant in CRC patients.^20^ Commensal bacteria *Fusobacterium nucleatum* was also found to increase intestinal tumorigenesis without aggravating colitis or inflammatory pathways,^21^ instead producing pro-tumorigenic formate^22^ and binding to proteins to mediate tumor growth.^23^ Others have recently found that increased *Lactobacillus reuteri* in the gut and its metabolite indole-3-lactic acid is associated with reduced CRC tumor burden.^24^ The presence of bacterial biofilms, particularly those containing bacterial phyla Bacteroidetes and Firmicutes, with significant populations of *Fusobacterium* spp. and the *Enterobacteriaceae* family, were also significantly associated with colorectal tumors.^15^ A follow-up study found that patients with familial adenomatous polyposis, an inherited condition that leads to polyps along the colon with a high incidence of CRC, had biofilms containing *E. coli* and *Bacteroides fragilis* that were capable of producing the endotoxins colibactin and *B. fragilis* toxin, respectively.^25^ On the other hand, *B. fragilis* has also been shown to be protective against CRC in *in vitro* models, as its capsular carbohydrate Polysaccharide A can inhibit proliferation of CRC cells and impair migration.^26^ Another group attempted to separate the bacterial signatures of CRC patients into clusters, finding that while some members of phyla Bacteroidetes and Firmicutes decreased, other members of these phyla increased,^27^ *Prevotella* and other bacterial genera thought to be oral commensals, such as *Fusobacterium*, *Porphyromonas*, *Anaerococcus*, *Parvimonas*, and *Granulicatella*, were also increased in CRC patients,^27^ Another group similarly attempted to separate CRC microbial signatures into metacommunities, with a metacommunity mainly containing *Bacteroides* spp. showing decreased prevalence and a metacommunity mainly containing oral bacteria, such as *Fusobacterium* spp., showing increased prevalence in CRC patients.^28^ In early-stage CRC, the genera *Fusobacterium*, *Parvimonas*, and *Gemella* were increased in abundance, while *Blautia* spp., *Faecalibacterium prausnitzii*, *Collinsella aerofaciens*, and *Alistipes putredinis* were among those that decreased in abundance, but none of these changes were significantly different from control tissue at later stages,^28^ suggesting that the microbial community may change as CRC progresses. Commensal bacteria belonging to Lachnospiraceae family, specifically *Ruminococcus gnavus* and *Blautia producta*, have been found to be protective against CRC, reducing colon tumor growth and enhancing immune activity in mouse models of CRC.^29^

Intratumoral bacteria have also been shown to modulate the progression of CRC. *E. coli* present within CRC tumors has been shown to disrupt the gut vascular barrier to create a new niche in the liver before CRC metastasis to this region.^30^ A meta-analysis of nearly 800 samples found that there were 94 bacterial species that differed between CRC patients and controls, including those belonging to genera *Fusobacterium*, *Porphyromonas*, *Parvimonas*, *Gemella*, and *Prevotella*, with a model built on these able to separate patients from controls with area under receiver operating curves (AUROC) scores above 0.8^31^ out of 1.0, which would be a perfect classifier. Further, CRC patients were found to have significantly higher abundance of genes coding for virulence factors, such as FadA, colibactin, and bile acid-inducible operon from some *Clostridium* spp., though no change was noted for *B. fragilis* toxin.^31^ In mouse models of CRC, administering the gut microbial metabolite gallic acid, even when the gut microbiome was not intact, was able to cause a malignant phenotype when a mutation in tumor suppressor p53 was present.^32^

A study analyzing nearly 900 patients that had been infected with protozoan parasite *Trypanosoma cruzi* and had developed an inflammatory condition known as megacolon found that no patient developed any form of CRC, suggesting that *T. cruzi* could be protective against CRC.^33^ Further studies on this parasite have found that its presence in vaccines strongly inhibits tumor development in rat models of colon and breast cancers by activating anti-tumor immune pathways.^34^ Fungi in the gut have also been implicated in CRC, with one group showing increased prevalence of many *Candida* spp., *Cyberlindnera jadinii*, and *Saccharomyces cerevisiae* in CRC patients, with the latter two possibly being due to diet.^35^ The authors note that they were able to separate tumors in being dominated by *Candida* spp. or *Saccharomyces cerevisiae*, with the former being associated with late-stage, metastatic disease and the latter associated with early-stage disease.^35^ Another group showed increases in *Malassezia* spp. and decreases in the orders Saccharomycetales and Pneumocystidales in patients.^36^ A model based on 14 fungal biomarkers was able to distinguish between controls and CRC patients, with AUROC scores above 0.7.^36^ A similar analysis of gut viruses found that the diversity of bacteriophages was significantly increased in CRC patients, with a model based on these changes distinguishing between controls and CRC patients with AUROC scores above 0.7.^37^ These virome changes were distinct between early-stage and late-stage patients and the genera *Betabaculovirus*, *Muvirus*, and *Punavirus* were among those associated with significantly lower survival rates.^37^ Another study of gut viruses in CRC patients saw no significant difference in diversity of viruses, but did note that the majority of viruses found were temperate bacteriophages with the families *Siphoviridae* and *Myoviridae* being highly associated with CRC.^38^

CRC has also previously been shown to result from increased inflammation in the gut and patients with inflamed colons due to the inflammatory bowel diseases (IBD) are at higher risk for developing CRC. Some microbes have been found to activate tumorigenesis indirectly through this method. For example, monocolonization with *E. coli* strain NC101 has been shown to cause inflammation and promote tumor formation.^39^ Further, similar *E. coli* strains have been increasingly found in patients with IBD and CRC.^39^ In addition, *Salmonella enterica* and its protein ArvA were found to be present intracellularly within colon tissue from CRC mouse models and were found to be increasingly present in patients with IBD.^40^ Another gut microbe, *Morganella morganii* and its production of DNA-damaging indolimines were found to be highly expressed in patients with IBD.^41^ In a mouse model of CRC, the inclusion of indolimine-producing *M. morganii* in a community of other human gut microbes significantly increased tumor burden.^41^ Patients with IBD have been found to carry an abundance of *Candida* spp., specifically *Candida albicans*,^42^ which has also been shown to be increased in CRC. While there have been many species that have been identified to be associated with CRC, additional studies in larger patient populations or meta-analyses are necessary to understand if these microbes are significantly different in all patients or simply a subset and more research is needed to learn more about the specific mechanisms these microbes may be utilizing and if those molecular pathways could be targeted.

### Other gastrointestinal cancers

Bacteria also was found to be highly expressed in pancreatic cancer samples, with phylum Proteobacteria dominating,^15,43^ though Bacteroides and Firmicutes were also abundant^43^. Further, *F. nucleatum*, which has been implicated in CRC, was also found to be highly prevalent in pancreatic cancer samples.^15,44^ *F. nucleatum* has also been shown to promote tumor progression in pancreatic ductal adenocarcinoma (PDAC), the most common form of pancreatic cancer, and migration in human cell lines.^45^ *Lactobacillus* spp., which have been shown to be protective in oral cancers, was decreased in patients with pancreatic cancer.^44^ Other microbes noted to be prevalent include those from the genera *Pseudomonas* and *Elizabethkingia*.^43^ In PDAC patients those with more diverse local microbiota had improved survival.^46^ Further, long-term survivors had tumors that were colonized with genera *Pseudoxanthomonas*, *Saccharopolyspora*, and *Streptomyces*, while short-term survivors had tumors that were colonized with classes Clostridia and Bacteroidea.^46^ A model based on the three genera, along with changes in *Bacillus clausii*, was found to predict long-term survival with an AUROC score above 0.97, with these microbes being implicated in immune activation pathways.^46^ Another group found that *Malassezia* spp. were increased in both human PDAC patients and mouse models of PDAC.^47,48^ Ablating the mycobiome was protective against PDAC tumors in mice and repopulation with *Malassezia* spp. resulted in increased tumor growth, due to differences in activation of multiple immune pathways. ^47,48^

The gut microbiome has also been implicated in other gastrointestinal cancers, including liver cancer and gallbladder cancer. Bile acids produced by gut microbes have been shown to decrease immunosurveillance, with antibiotic treatment in mouse models of liver cancer able to inhibit liver tumor growth.^49^ In mice that were exposed to chemical or infectious carcinogens, colonization with *H. hepaticus* was sufficient cause formation of hepatocellular carcinoma, the most common form of liver cancer.^50^ Gallbladder cancer patients have been found to be more likely to have antibodies against *Salmonella enterica* serovar Typhi, despite the bacteria itself not being found in blood, tissue, or fecal samples, though how these antibodies may be related to carcinogenesis is not well understood.^51^ Following multiple meta-analyses, researchers have also noted an association between *Heliobacter* spp. and different biliary tract cancers, including cancers of the bile ducts and gallbladder.^52^ Other microbial changes, including increases in families *Fusobacteriaceae*, *Enterobacteriaceae*, and *Pseudomonadaceae*, have also been consistently noted across multiple studies.^52^

### Oral cancers

Various oral cancers have been shown to be associated with changes in the oral microbiome. An analysis of 121 oral cancer patients found that *Dialister* spp. were significantly increased, while bacteria belonging to orders Actinomycetales and Lactobacillales, *Streptococcus* spp., and *Corynebacterium* spp. were significantly decreased.^53^ Following total tooth loss, another known risk factor for developing oral cancer, the oral microbiome changed more extensively, with 122 clades of bacteria decreasing in abundance.^53^ Other known risk factors of oral cancer, including smoking, HPV status, and periodontal disease, were also associated with significant changes in the oral microbiome.^53^ With improvements in identification methods, others have found that that in patients with OSCC, genera *Firmicutes*, *Bacteroidetes*, *Proteobacteria*, and *Actinobacteria* were more abundant, with *Capnocytophaga* spp. also being associated with late-stage tumors.^54^ In mouse models of OSCC, *P. gingivalis* and *F. nucleatum* have been shown to directly interact with oral epithelial cells and stimulate tumorigenesis by stimulating proliferation and affecting key pathways.^55^ Another analysis of oral cancer patients found that the phyla Firmicutes and Actinobacteria significantly decreased in the initial cancer samples and validation samples, while the Fusobacteriota phylum (mainly *F. nucleatum*) was significantly increased.^56^ *Streptococcus* spp. were also significantly decreased in pre-cancerous samples.^56^

In tongue cancer patients, Firmicutes significantly increased, while Bacteroidetes and Fusobacteria significantly decreased, with the genera *Streptococcus*, *Actinomyces*, *Rothia*, *Corynebacterium*, *Enterococcus*, and *Micrococcus* being the most significantly increased.^57^ While oral fungal diversity was not significantly different in tongue cancer patients, there was a significant decrease in the fungal richness, though the fungal reads present were much less numerous than bacterial reads.^57^ *C. albicans* has also been found to be often present in oral cancer patients, with possible mechanisms for how they may directly influence carcinogenesis previously being reviewed,^58^ though more research is needed to understand which of these possible pathways the fungi may be using.

### Breast cancer

While breast tissue was not originally thought to have its own microbiome, there has been increasing research showing that microbes are present in both normal breast tissue and breast cancer tissue. Total bacterial DNA was found to be reduced in breast tumor tissue and bacterial DNA load was found to be inversely correlated with more advanced disease stages,^59^ suggesting that cancer may disrupt the commensal network in this region. Bacteria was found to be expressed in over 60% of breast cancer samples and occurred intracellularly in breast cancer cells and immune cells, with the bacteria found in breast tumor samples being more diverse than other cancer types and showing specific variations based on receptor status.^15^ Specifically, researchers were able to isolate live bacteria from the phyla Proteobacteria, Firmicutes, and Actinobacteria in breast tumors.^15^ Notably, *F. nucleatum*, previously implicated in CRC, was found to also be more abundant in breast tissue^15^ and has been shown to colonize mammary tissue to promote tumor growth and metastasis.^60^ Bacterial species *Methylobacterium radiotolerans* has also been found to be increased in tumor tissue, while *Sphingomonas yanoikuyae* was decreased.^59^ Fungal genera *Malassezia* has also been shown to be abundant in breast tumors.^35^

The impact of the local microbiome in breast cancer has also been shown in mouse models, with a depletion in intratumoral bacteria being associated with less lung metastases, with *Staphylococcus* spp. and *Lactobacillus* spp. being associated with an increase in metastatic tumors.^61^ Gut microbes have also been shown to impact the development and progression of breast cancer. In mouse models of breast cancer, a gavage with *H. hepaticus* has been shown to increase mammary tumor burden, inflammation in mammary tissue, and neutrophil load.^62^ Through additional research on the normal microbiome of breast tissue and the changes that occur in this with breast cancer, microbes may serve as new targets for breast cancer treatments and prevention methods. Additionally, further research is needed to understand how prevalent these specific microbial changes are across patient populations.

### Lung cancer

The microbiota of lung tissue can be impacted by proximal microbial communities in the oral cavity, nasal cavity, and gastrointestinal tract and has been shown to be implicated in lung cancers. Lung cancer patients were found to have lower bacterial diversity, with the genera *Acidovorax*, *Klebsiella*, *Rhodoferax*, and *Anaerococcus* significantly enriched in smokers with squamous cell lung carcinoma.^63^ Further, the presence of genera *Acidovorax*, *Klebsiella*, *Rhodoferax*, *Comamonas*, and *Polarmonas* was highly associated with p53 mutations.^63^ Researchers have seen increases in members of the Proteobacteria phylum in lung cancer patients, but the significance of this association varies by study. ^15; 63^ Fungal genera *Blastomyces* was also found to be increased in patients with squamous cell lung carcinoma.^35^ Smokers with lung cancer were found to have a distinct microbiome, with an increased abundance of the phyla Proteobacteria, Actinobacteria, and Cyanobacteria and decreased abundance of the phylum Firmicutes.^15^

In a mouse model of lung adenocarcinoma, a type of NSCLC, local bacterial diversity decreased, while the bacterial load increased, with *Herbaspirillum* spp., the family *Sphingomonadaceae*, *Aggregatibacter* spp., and *Lactobacillus* spp. being significantly enriched.^64^ In NSCLC patients, genera *Veillonella, Prevotella*, and *Streptococcus* were found to be more strongly associated with more advanced disease, while genus *Flavobacterium* was more strongly associated with earlier stages.^65^ Disrupting the lung microbiome with *Veillonella parvula* in a lung cancer mouse model was found to cause dysbiosis, leading to decreased survival and increased tumor burden.^65^ Lung cancer is rather unique, as it can easily be affected by its own microbiome in the lung, as well as the proximal communities of other regions (oral, nasal, gastrointestinal), and as a result, could be targeted through the modulation various microbiomes. Further research is needed to better understand the mechanisms of how changes of these different microbiomes affect lung cancer and how translatable these changes are across patient populations and different lung cancer types.

### Urogenital and reproductive cancers

While the urinary and reproductive tracts have microbiomes that are distinct from other regions, these local microbiomes have also been implicated in urogenital and reproductive cancers. In a mouse model based on inflammation and susceptible to prostate cancer, gut bacteria *H. hepaticus* was shown trigger carcinogenesis.^66^ Further, an injection of lymph node cells from mice infected with *H. hepaticus* was able to trigger prostate carcinogenesis in other mice of this model and neutralizing inflammation was able to halt this transmissibility.^66^ Sequencing of voided urine from patients with and without prostate cancer found that samples mainly contained genera *Corynebacterium*, *Staphylococcus*, and *Streptococcus*, though the microbial reads were low.^67^ Bacterial species *Streptococcus anginosus*, *Anaerococcus lactolyticus*, *Anaerococcus obesiensis*, *Actinobaculum schaalii*, *Varibaculum cambriense*, *Propionimicrobium lymphophilum*, and *Ureaplasma* spp. were all found more often in prostate cancer patients, with most of these having been previously implicated in other urogenital infections,^67^ showing that pro-inflammatory microbes are more prevalent in patients with prostate cancer. However, further research is still needed to understand if these microbes are causing cancer through sustained local inflammation or some other method, such as the release of genotoxic factors. Similarly, the urinary microbiome has also been implicated in bladder cancer, with studies showing the increased abundance of the genera *Streptococcus*, *Fusobacterium*, *Acinetobacter*, *Anaerococcus*, *Sphingobacterium*, *Herbaspirillum*, *Porphyrobacter*, and *Bacteroides*, though the number of patients analyzed in these studies is low, there seems to be considerable variation between studies, and these studies utilize voided urine, which has been previously described to be not adequately representative of the bladder microbiome.^68^ Additional studies with larger patient populations may be useful to understand if the different microbes identified are specific to certain patient populations or if other factors, such as location or diet, may also be affecting the bladder microbiome.

The cervicovaginal microbiome has also been shown to affect the development and progression of ovarian, endometrial, and cervical cancers. While the normal healthy cervicovaginal microbiome mainly contains *Lactobacillus* spp., resulting in a low vaginal pH, in ovarian cancer patients, non-*Lactobacillus* species made up a majority of the microbiome, with this association being stronger in younger patients.^69^ Another study showed that ovarian cancer samples had a distinct microbiome, with changes in viruses, bacteria, fungi, and parasites. Viral signatures showed the presence of families *Retroviridae*, *Hepadnaviridae*, and *Papillomaviridae*, all of which contain known oncomicrobes.^70^ Bacterial signatures showed the presence of *Shewanella* spp. and *Pediococcus* spp. among others and fungal signatures showed 18 species that were expressed only in cancer tissues, including *Cladosporium*, with few fungal species noted in controls, suggesting that the fungal signatures could be the best microbial biomarkers.^70^ The authors do also note that there are also differences in parasite signatures, with *Dipylidium*, *Trichuris*, and *Leishmania* present in all cancer samples,^70^ with these parasites known to be more prevalent in non-Western countries. In endometrial cancer patients, multiple phyla were enriched, including Firmicutes, Spirochaetes, Actinobacteria, Bacteroidetes, and Proteobacteria, with *Atopobium vaginae* and *Porphyromonas somerae* showing the highest association with disease, especially when a high vaginal pH was present.^71^

In addition to human papillomavirus (HPV), which has been shown to directly cause cervical cancer as one of the 11 IARC oncomicrobes, other changes in the microbiome have also been noted. The predominance of *A. vaginae* has been implicated in cervical cancer.^72^ Vaginal dysbiosis has been associated with the presence of HPV, bacterial vaginosis (overgrowth of bacteria in the vagina), and cervical cancer.^73^ *Sneathia* spp., one of the possible causes of bacterial vaginosis, has also been shown to be enriched in pre-cancerous patients.^74^ Increased genital inflammation and changes to the vaginal microbiome, mainly the decrease in *Lactobacillus* spp., has been shown to drive cancer formation when coupled with HPV infection. ^74; 75^ As *Lactobacillus* spp. maintain the low pH of the vagina, increased vaginal pH has also been linked to increased cancer risk.^74^ There have also been differences noted between high-grade and low-grade cervical cancer patients, with high-grade patients having increased *Sneathia sanguinegens*, *Anaerococcus tetradius*, and *Peptostreptococcus anaerobius* and decreased *Lactobacillus jensenii* compared to low-grade patients,^76^ suggesting that the dysbiosis may worsen as the cancer develops and progresses, though the mechanism for these changes alongside cancer progression would need to be studied further. Other studies have also shown that microbial abundance in cervical squamous cell carcinoma predicts survival better than clinical factors, with survival outcomes strongly negatively correlated with the abundance of the genera *Lactobacillus* and *Chlamydia*.^77^

### Other cancers

B-cell acute lymphoblastic leukemia (ALL) has been previously associated with infectious agents. Mouse models of B-cell ALL have shown that antibiotic treatment in mice predisposed to leukemia was sufficient to trigger the disease, even without other infectious stimuli.^78^ These mice also had lower levels of genera *Alistipes* and *Oscillospira* in their gut microbiome, compared to mice that were not predisposed to leukemia.^78^ In patients with ALL, gut microbiome changes have also been noted, with lower diversity, and a lower abundance of genera *Anaerostipes*, *Coprococcus*, *Roseburia*, and *Ruminococcus*,^79^ though additional studies are needed to better understand these changes.

Recently, the intratumoral microbiota was also shown to be associated with adrenocortical carcinoma, a rare malignancy of the adrenal glands, with increased bacterial diversity and a prevalence of genera *Bacteroides* and *Streptomyces*.^80^ These microbes also showed a correlation with survival outcomes, with AUROC scores consistently above 0.8 and inflammatory and cell cycle pathways also being implicated.^80^ This is consistent with a previous study which showed that microbial features were able to better predict survival outcomes long-term compared to prognostic markers in patients with adrenocortical carcinoma.^77^

## The impact of microbiome on cancer diagnostics

Early diagnosis of cancer has been shown to be linked to lower incidence of negative outcomes and higher incidence of successful treatment. The current methods of cancer diagnosis vary in their invasiveness, with many diagnoses still requiring invasive biopsies for confirmation. For this reason, research has focused on finding less invasive methods that still maintain a high degree of sensitivity (including cancer cases) and specificity (excluding non-cancer cases). As previously mentioned, this was also sometimes conveyed as an AUROC score, with a value of 1.0 representing a perfect classifier with 100% specificity and 100% sensitivity. Recent research has focused on how microbiomes could be used as a less invasive method of diagnosis (either directly or indirectly related to cancer), as saliva samples, stool samples, and plasma samples can be more easily obtained than other methods currently used for diagnosis.

### Salivary microbiota

One of the most researched diagnostic methods is the salivary microbiota, which can be studied through noninvasive saliva samples. The salivary microbiota has been found to be associated with cancers of multiple regions, including the mouth, pancreas, and lungs. For patients with oral squamous cell carcinoma (OSCC), the combination of bacterial species *Capnocytophaga gingivalis*, *Prevotella melaninogenica*, and *Streptococcus mitis* in the salivary microbiome was found to serve as a diagnostic indicator with a sensitivity of 80% and specificity of 83% compared to healthy controls^81^. While these three bacterial species are all commensals, they were found to be significantly elevated in cancer patients.^81^ An additional recent study has suggested that *C. gingivalis* could be a tumor promoter,^82^ but the possible mechanisms for the other two species remain unknown.

For patients with pancreatic cancer, the combination of bacterial species *Neisseria elongata* and *S. mitis* in the salivary microbiome was found to serve as a diagnostic indicator with a sensitivity of 96% and specificity of 82% compared to healthy controls, while the combination of bacterial species *Granulicatella adiacens* and *S. mitis* could distinguish between pancreatic cancer patients and patients with pancreatitis with a sensitivity of 86% and specificity of 53%.^83^ These are all also commensal oral microbes, yet in these cancer patients, *N. elongate* and *S. mitis* were found to be significantly decreased and *G. adiacens* was significantly increased.^83^ It has been proposed that these bacteria may be involved with systemic inflammation, but evidence on the interactions between these bacteria and cancer development remains limited. An additional larger study found that *S. mitis* was significantly decreased in pancreatic cancer patients and noted that another species, the oral pathogen *Porphyromonas gingivalis*, was also significantly decreased, but did not discuss its possible use as diagnostic marker.^84^ A more recent study utilizing shotgun metagenomic sequencing and 16S rRNA amplicon sequencing of saliva samples of patients with PDAC noted that there was no significant difference in previously reported associations of oral microbes, including *P. gingivalis*, *N. elongate*, and *S. mitis*,^85^ so it is unclear how applicable these microbes are as a diagnostic indicator.

For patients with lung cancer, the combination of two bacterial genera, *Capnocytophaga* and *Veillonella*, in the salivary microbiome was found to serve as a diagnostic indicator with a sensitivity of 85% and specificity of 87% between patients with squamous cell lung carcinoma and healthy controls and with a sensitivity of 79% and specificity of 90% between patients with lung adenocarcinoma and healthy controls^86^. Similar to the previously mentioned, these genera are oral commensals, but were significantly higher in abundance in both of lung cancer populations.^86^ Another study utilizing different methods found that bacterial genera *Acidovorax* and *Veillonella* in combination were the best biomarkers for squamous cell lung carcinoma with a sensitivity of 80% and specificity of 89%, whereas *Capnocytophaga* alone could detect lung adenocarcinoma with a sensitivity of 73% and specificity of 85%.^87^ These three genera have been noted across multiple studies as being biomarkers specific to lung cancer, with other studies noting that *Veillonella* could play a role in carcinogenesis by altering inflammatory genes and affecting the prevalence of other microbes, that *Acidovorax* may be selected for by smoking behaviors and may form biofilms and suppress immunity, and that *Capnocytophaga* may induce long-term inflammation.^88^

A major limitation of utilizing the salivary microbiota as a diagnostic is that it is often unclear if differences in microbes is a cause or result of cancer. That is, these bacteria may have a fold change following cancer development and thus, would be less useful for diagnosing cancerous formation at an earlier stage, though further research is needed to tease apart this association. Additionally, the oral microbiome can be significantly different depending on the age, race/ethnicity, diet, and lifestyle of patients, and so, larger patient populations need to be studied to ensure a high degree of diagnostic sensitivity and specificity across all populations.

### Fecal microbiota

As with the oral microbiome, the fecal microbiome has also been studied thoroughly and can be studied using noninvasive methods of collection with stool samples. The majority of research on the fecal microbiome as a diagnostic biomarker has been concentrated on CRC. One study found that a bacterial gene marker from the recently characterized *Lachnoclostrium* spp. was significantly increased in colorectal adenomas and had a sensitivity of 48% and specificity of 79% for adenomas.^89^ By combining this marker with additional bacterial species *F. nucleatum*, *Clostridium hathewayi* (now *Hungatella hathewayi)*, and *Bacteroides clarus*, sensitivity and specificity were improved to 94% and 81%, respectively, for CRC.^89^ Researchers have also used *Clostridium symbiosum* alone as a biomarker for CRC, finding that it had a sensitivity of 80% and specificity of 55%, making it a more sensitive biomarker than the well-characterized cancer-inducing pathogen *F. nucleatum*.^90^ A model analyzing *C. symbiosum* had AUROC scores of 0.74 for early-stage CRC and 0.76 for all CRC stages, both of which are an improvement over fecal immunochemical test (FIT), one of the current stool DNA-based CRC tests.^90^ By analyzing *C. symbiosum* abundance and FIT together, these AUROC scores were improved further to 0.80 and 0.87,^90^ suggesting that fecal microbial biomarkers in combination with other existing diagnosis methods may be a viable future direction. Another study utilizing probe-based qPCR found that the combination of *Prevotella copri*, *Gemella morbillorum*, *Parvimonas micra*, *Cetobacterium somerae*, and *Pasteurella stomatis* predicted CRC occurrence with a sensitivity of 68% and specificity of 89%.^91^

In order to understand if these markers were applicable across diverse populations, researchers have also carried out a meta-analysis of fecal metagenomes for CRC. Utilizing shotgun metagenomics of eight previous study populations, researchers found that a set of 29 bacterial species were enriched, including *F. nucleatum*, *P. micra*, *G. morbillorum*, *Prevotella* spp., and *Clostridium* spp.^31^ The model trained with these metagenomic data sets was able to successfully differentiate between CRC patients and controls, with AUROC scores ranging between 0.71 and 0.92.^31^ A similar study utilizing shotgun metagenomics of five previous study populations also noted *F. nucleatum*, *P. micra*, *C. symbiosum*, and *G. morbillorum*, as four of six bacterial biomarkers that were present among multiple cohorts.^92^ The model trained on these metagenomic data sets was also successful, with AUROC scores above 0.8.^92^ Additional analysis found that trimethylamine synthesis was increased, likely due to a species of the family *Lachnospiraceae* that had not yet been characterized and *H. hathewayi*, among others,^92^ These metagenomic studies do help to show that these microbial biomarkers are consistent across patient populations and that external factors are less likely to confound the models. This said, whether these microbial changes are occurring upstream or downstream of CRC development still remains to be determined.

While less common, the fecal microbiome has also been used to study biomarkers for other cancer types. For example, a 16S rRNA analysis of the gut microbiome of lung cancer patients found that 13 bacterial genera were decreased, while 11 bacterial genera were increased.^93^ Further, using a model based on 9 of these genera, including *Bacteroides* and *Clostridium*, researchers were able to predict lung cancer diagnoses with an average AUROC score of 0.76.^93^ However, additional research is needed to understand why these genera may be gut biomarkers for lung cancer and if this model is still accurate across larger patient populations.

### Plasma cell-free DNA

Unlike the previously described methods of analyses from microbially-rich regions, there has recently been a growing interest in how cell-free DNA from human plasma could also be used in cancer diagnosis. Diagnostic procedures based on cell-free DNA, also called liquid biopsies, have increased rapidly, from their original use in prenatal testing to being used for other health conditions. In fact, the cobas EGFR Mutation Test was approved by the US Food and Drug Administration (FDA) in 2016, as one of the first such tests used for cancer, using plasma cell-free DNA to analyze mutations in the epidermal growth factor receptor (EGFR) gene to improve treatments for patients with non-small cell lung cancer (NSCLC),^94^ with another similar EGFR test, Guardant360 CDx, being approved by the FDA in 2021,^95^ Another liquid biopsy test, FoundationOne Liquid CDx, was approved by the FDA in 2020 and tests for over 300 genes that have been implicated in NSCLC, prostate cancer, ovarian cancer, and breast cancer as a companion diagnostic tool.^96^

However, these tests focus on utilizing circulating tumor DNA present in the plasma. As previous research has shown that microbes can be associated with cancer, as we have discussed, recent studies have analyzed if circulating microbial DNA sequences may also be present in the plasma and if these sequences could be used in diagnostic tests. A next-generation sequencing analysis of three patients with early-onset breast cancer found that microbial reads were present in plasma, with the majority being from bacteria, though some were also from fungi and viruses.^97^ Bacterial reads in plasma were found to be distinctly different between the cancer patients and control group, with the controls having more *Acinetobacter* spp. reads, while cancer patients had more *Pseudomonas* spp. and *Sphingomonas* spp. reads, though the authors do mention that further research with a larger cohort would be needed to draw any conclusions.^97^ Additional analyses have found that microbial signatures in the plasma change even when patients with a variety of cancer types do not have genetic mutations, with authors also mentioning that microbial reads could be present in blood fractions other than the plasma.^98^ A model trained on these signatures could distinguish patients with 20 different cancer types from each other and healthy controls, with AUROC scores consistently above 0.85.^98^ Moreover, this model was able to still distinguish between cancer types when analyzing samples from patients with stage I and stage II cancers of various origins, with AUROC scores above 0.80.^98^ The same group also analyzed how fungal reads in the blood plasma could also be predictive of the 20 cancer types, with the model based on the fungal reads averaging AUROC scores of 0.85, while the model based on bacterial reads averaged AUROC scores of 0.90.^99^ By combining both sets of data, average AUROC scores improved slightly to 0.92,^99^ suggesting that fungi also have notable changes associated with cancer and that a combination of different types of microbial reads could be used to detect cancer, even if genomic changes are not present. While recent, these successes in using microbial reads from plasma cell-free DNA have shown that this method of liquid biopsies may be a viable diagnostic tool for diagnosing cancer, particularly at an earlier stage when other less invasive tools may be less accurate, though further studies are needed to corroborate and improve this method.

While there has been some success in utilizing various microbiomes as diagnostic tools to detect cancer experimentally (Table 1), we do note that it is likely that such microbiome-based diagnostic methods will be used alongside other existing methods, such as imaging or biopsies, for the foreseeable future, rather than an independent diagnostic approach. However, these methods could be particularly important to diagnose cancer at earlier stages and lessen the number of invasive diagnostics patients would need to go through. As the field further understands the complex interactions between microbiomes and cancer both spatially and functionally, we predict that such diagnostic methods based on the microbiome could become independent approaches in the future.

**Table 1.** Summary table of selected diagnostic methods and their diagnostic ability based on area under receiver operating curve (AUROC) scores, as previously described.

Diagnostic method	Cancer type	Microbial marker(s)	AUROC score (out of 1.0)	Reference
Salivary microbiome	pancreatic	*N. elongata* and *S. mitis*	0.90	Farrell et al., 2011^83^
Salivary microbiome	squamous cell (lung)	*Capnocytophaga* and *Veillonella*	0.86	Yan et al., 2015^86^
Fecal microbiome	colorectal	*C. symbiosum*	0.73	Xie et al., 2017^90^
Fecal microbiome	colorectal	*F. nucleatum*	0.86	Liang et al., 2020^89^
Fecal microbiome	lung	various bacteria	0.76	Zheng et al., 2020^93^
Plasma cell-free DNA	various (20) types	various bacteria	0.90	Poore et al., 2020^98^
Plasma cell-free DNA	various (20) types	various fungi	0.80	Narunsky-Haziza et al., 2022^99^
Plasma cell-free DNA	various (20) types	various bacteria + fungi	0.92	Narunsky-Haziza et al., 2022^99^

## The effect of microbiome on cancer therapies

As a previous review has discussed,^100^ cancer has been documented as far back as 3000 B.C.E. Around 150 C.E., Galen described surgical strategies for removing tumors, particularly for tumors that were superficial. The birth of radiotherapy began around the early 20^th^ century, with many scientists realizing that radiation could be used to kill tumors. Following the end of the war, further studies on compounds developed for chemical warfare during World War I led to the discovery of nitrogen mustard, the first chemotherapeutic agent. Despite being over a century old, all three of these treatment methods (surgery, radiotherapy, and chemotherapy) are still currently used as primary first-line treatments. Hematopoietic stem cell transplants for blood-related conditions began in the 1950s, with a particular focus on preventing the complication of graft-versus-host disease (GvHD),^101^ with the first successful hematopoietic transplant as a treatment for blood cancers taking place in the mid-1970s. In the 1980s, the emergence of targeted therapies focused on key genes resulted in drugs that inhibited the growth and hormonal pathways upregulated by cancer. These therapies could preferentially target cancer cells, unlike the less selective nature of chemotherapeutic agents. Targeted therapies have been utilized in the treatment of many types of cancer, with hormonal therapies being particularly impactful for reproductive and urogenital cancer treatment. In the past decade, targeted therapies have transitioned their focus to tumor antigens and T cell receptors, utilizing the immune system to treat cancer. While immunotherapy is usually not a first-line treatment, it has been shown to significantly improve outcomes in patients that have not responded to other treatments. Additional recent advances in immunotherapy have included T cells with chimeric antigen receptors (CAR-T), a method of targeting cancer cells using the patient’s own cells that have been genetically modified, which has shown great success. There have been proposals to utilize newly developed methods, such as CRISPR/Cas9, to find new ways to treat cancer, though these are still under study.

Microbes have continued to be associated with many of these cancer therapies (Table 2), though additional studies are needed to better understand the mechanisms by which these microbes are involved. The majority of studies focused on cancer treatments and microbes have found that certain microbes are associated with changes in host immune responses, though other interactions, such as changes to protective bacterial metabolites or increased drug resistance, have also been noted. Further, by better understanding the interactions between microbes and cancer, new microbial targets for cancer treatments may also emerge.

**Table 2.** Summary table of representative microbes known to affect cancer therapies, as described further below.

Cancer therapy	Cancer type(s)	Microbe (microbiome)	Implication of microbes	Reference
Radiotherapy	melanoma, lung cancer, and cervical cancer models	Gram-positive bacteria (gut)	Changes to immune responses and tumor growth inhibition	Uribe-Herranz et al., 2020^102^
Radiotherapy	breast cancer	fungi (gut)	Changes to immune responses	Shiao et al., 2021^103^
Chemotherapy (cyclopho-sphamide)	melanoma and sarcoma models	*Lactobacillus johnsonii* and *Enterococcus hirae* (gut)	Changes to immune responses	Viaud et al., 2013^104^
Chemotherapy (gemcitabine)	pancreatic cancer	*Gammaproteobacteria* (tumor)	Inactivation of drug by bacterial metabolism	Geller et al., 2017^105^
Chemotherapy (dacarbazine)	melanoma lung metastases model	*Lactobacillus rhamnosus* (lung)	Changes to immune responses	Le Noci et al., 2018^106^
Immunotherapy (anti-CTLA-4)	melanoma model	*Bacteroides fragilis*, *Bacteroides thetaiotaomicron*, *Burkholderia cepacia* (gut)	Changes to immune responses	Vétizou et al., 2015^107^
Immunotherapy (anti-PD-1)	melanoma	*Faecalibacterium* spp. (gut)	Changes to metabolic functions and immune responses	Gopalakrishnan et al., 2017^108^
Immunotherapy (anti-PD-L1)	melanoma model	*Bifidobacterium* spp. (gut)	Changes to immune responses	Sivan et al., 2015^109^
Immunotherapy (CpG-oligonucleotide)	lymphoma, colon cancer, and melanoma models	*Alistipes* spp., *Ruminococcus* spp., *Lactobacillus* spp. (gut)	Changes to immune responses and treatment-related cytotoxicity	Iida et al., 2013^110^
Immunotherapy (CAR-T)	B-cell lymphoma	*Bacteroides* spp., *Ruminococcus* spp., *Eubacterium* spp., and *Akkermansia* spp. (gut)	Changes to immune responses	Stein-Thoeringer et al., 2023^111^
Allo-HSCT	various blood cancers	*Eubacterium limosum* and other *Eubacteriaceae* (gut)	Changes to graft-vs-tumor effects	Peled et al., 2017^112^
Allo-HSCT	various blood cancers	*Lachnospiraceae* and *Ruminococcaceae* (gut)	Changes in protective metabolites	Payen et al., 2020^113^

### Radiotherapy

Radiation therapy, also called radiotherapy, is one of the oldest treatments for cancer and mainly works by damaging cancer DNA to trigger cell death. This therapy is most often given locally, though it can also be given in conjunction with other therapies. It has been well-documented that gastrointestinal distress is a common side-effect of radiation therapy, even when this region is not irradiated, suggesting that gut microbes may somehow be implicated.

For this reason, most of the research on the interaction of microbes and radiation has focused on the gut microbiota. Researchers found that eradicating Gram-positive bacteria through antibiotics improved the antitumor effects of radiation in melanoma, lung cancer, and cervical cancer mouse models.^102^ Further, reintroducing the short-chain fatty acid (SCFA) metabolite sodium butyrate, normally produced by Gram-positive bacteria, nullified the improvement in radiotherapy seen with antibiotic treatment.^102^ Complete depletion of gut bacteria has also been shown to reduce the efficacy of radiation treatment in breast cancer and melanoma mouse models, whereas depletion of gut fungi improved this efficacy, due to opposing effects on immune recruitment.^103^ Similarly, a higher concentration of fungal sensor Dectin-1 was found to be associated with worse survival outcomes in breast cancer patients and fungal-free mice were found to have slower tumor growth following radiation treatment.^103^ Researchers have also found that mice that did not respond to radiation had different microbiota signatures to those that did, with the families *Enterococcaceae* and *Lachnospiraceae* being the most enriched bacteria in responders.^114^ An analysis of leukemia patients exposed to whole-body radiation showed that patients with less gastrointestinal symptoms also had significantly higher abundances of *Enterococcaceae* and *Lachnospiraceae*.^114^ While *Lachnospiraceae* did protect against these symptoms, it did not reduce the efficacy of radiation treatment in melanoma and lymphoma mouse models.^114^ Further metabolism experiments and metabolomics showed that SCFAs and tryptophan metabolites produced by these bacteria were significantly associated with radioprotection.^114^ While there has not been as much research done on the role of the microbiome in response to irradiation compared to other cancer treatments, these studies support the notion that the microbiome, specifically the gut microbiota, could impact patient responsiveness to this treatment and suggests a need for further research on this topic.

### Chemotherapy

Chemotherapy broadly refers to drugs that treat cancer through chemical means, often by impairing mitosis. The sensitivity of different cancers to chemotherapy varies widely. The goal of chemotherapy is often to damage or stress cancer cells until apoptosis is triggered, with some chemotherapeutic drugs also eliciting immune responses. This form of therapy is given systemically, so normal cells that also divide rapidly can also be affected. The FDA has approved hundreds of drugs as chemotherapeutic agents.

The gut microbiome was the first region to be implicated in chemotherapeutic efficacy, as with this treatment being given systemically, it was likely to affect these microbes. Research on this topic began with efficacy being measured following antibiotic treatment. Neutralizing the gut microbiome through antibiotics led to a reduced effect of the chemotherapeutic drugs cisplatin and oxaliplatin on lymphoma and colon cancer mouse models, due to a decrease in reactive oxygen species production, suggesting that an intact commensal microbiome is necessary for these platinum-based therapies.^110^ Antibiotic treatment also reduced efficacy of oxaliplatin in a subcutaneous CRC mouse model, with further experiments with fecal microbiota transfer (FMT) showing that *Paraprevotella clara* was associated with mice that did not respond to oxaliplatin, while *B. fragilis* was associated with those that did respond.^115^ Once it was understood that the gut microbiome could affect drug efficacy, researchers began to study the effects of chemotherapy on gut bacteria that could be easily cultured. Chemotherapeutic drug cyclophosphamide led to gut microbes, mainly *Lactobacillus johnsonii* and *Enterococcus hirae*, translocating to the spleen and lymph nodes, leading to an immune cascade in mouse models of melanoma and sarcoma^104^. Further research from this group showed that the presence of *E. hirae* and *Barnesiella intestinihominis* in the gut could promote cyclophosphamide activity and restore the efficacy of cyclophosphamide following antibiotic treatment.^116^ With improvements in omics technologies, researchers began to examine these microbes more closely, focusing on the individual metabolites produced by these microbes. Gut microbiome metabolite indole-3-acetic acid was found to improve therapeutic efficiency of the combination chemotherapy FOLFIRINOX (folinic acid, fluorouracil, irinotecan, oxaliplatin) in patients with metastatic PDAC, as it led to an accumulation of reactive oxygen species and decreased autophagy of cancer cells.^117^

However, it is not only the gut microbiome that has been found to affect chemotherapeutic efficacy. There has been increased interest in how local bacteria near or within the tumor microenvironment may not only affect therapeutic efficacy, but also contribute to chemoresistance in patients. The human commensal *E. coli* was found to upregulate the cytotoxicity of tegafur and mercaptopurine, while downregulating the cytotoxicity of gemcitabine, doxorubicin, and mitoxantrone in *in vitro* studies, as *E. coli* was able to metabolize these drugs.^118^ Further testing of gemcitabine with *E. coli* in a colon cancer mouse model showed that its presence impaired the drug’s antitumor activity.^118^ Others have found that gemcitabine could be metabolized to its inactive form by intratumoral bacteria, especially *Mycoplasma hyorhinis* and members of the class *Gammaproteobacteria*, including *E. coli*, resulting in cancer cells becoming resistant to gemcitabine.^105^ A co-culture of commensal *F. nucleatum* with colon cancer cell lines reduced apoptosis of these cancer cells when exposed to oxaliplatin and 5-flurouracil and led to oxaliplatin resistance in a colon cancer mouse model, mainly due to changes to autophagy and innate immune pathways.^119^ Both aerosolized antibiotics and a probiotic of aerosolized *Lactobacillus rhamnosus* improved the effect of chemotherapeutic drug dacarbazine,^106^ showing that the lung microbiota can be manipulated and that the lung microenvironment can also affect chemotherapeutic efficacy. These studies support the idea that microbes from other regions, local tissues, and the tumor microenvironment itself can all affect chemotherapy efficacy, and further research understanding these interactions may improve patient response to different chemotherapeutic drugs.

### Immunotherapy

The interest in cancer immunotherapy has risen over the past few decades, following improvements in our understanding of the immune system and how it can be used to fight cancer. As cancer cells often have unique tumor antigens, immunotherapy can work by binding to these and triggering the immune system to recognize and kill or inhibit cancer cells. CAR-T also works through this method, as the genetically-modified T cell receptors can better target tumor antigens. There are currently six CAR-T therapies that have been approved by the FDA for various forms of lymphoma, B-cell acute lymphoblastic leukemia (B-cell ALL), and multiple myeloma,^120^ Immunotherapy has also been used to stimulate the immune system by targeting immune checkpoints downregulated by cancer cells, rather than directly targeting the cancer cells themselves. The current FDA-approved immune checkpoint immunotherapies target the immune checkpoints CTLA-4, PD-1, or PD-L1, though additional checkpoint targets are currently being explored.^121^

Anti-CTLA-4 immunotherapy has been shown to require the presence of the gut microbiota to be most effective. Following antibiotic treatment and recolonization with *B. fragilis*, *Bacteroides thetaiotaomicron*, *Burkholderia cepacia*, or a combination led to improved anti-CTLA-4 treatment efficacy, with *B. fragilis* also being implicated in anti-cancer immune response^107^. Further experiments using FMT from patients showed that the abundance of *B. fragilis* was associated with smaller tumor sizes^107^. Colitis is a common side-effect of immunotherapies, so the gut microbiome has also been studied in this context. Patients that were colitis-free following anti-CTLA-4 immunotherapy had a higher abundance of bacteria belonging to the families *Bacteroidaceae*, *Rikenellaceae* and *Barnesiellaceae*, with a model trained on these reads able to categorize patients with a sensitivity of 70% and specificity of 100%.^122^ Another study analyzing fecal samples of patients following anti-CTLA-4 immunotherapy found that responders were enriched in *Faecalibacterium* spp. and other members of the phylum Firmicutes, but these microbes were also associated with colitis phenotypes^123^. Another group similarly found that genera *Faecalibacterium* and *Gemminger* were associated with progression-free survival of at least six months following anti-CTLA-4 immunotherapy, and that while genera *Faecalibacterium*, *Lachnospiracae*, and *Gemminger* were increased and genus *Prevotella* was decreased in patients with colitis, this was not significant^124^. Further, enrichment of the gut microbe *F. prausnitzii* led to better outcomes, which were attributed to production of SCFA metabolite butyrate.^124^ An abundance of *F. prausnitzii* was associated with lower butyrate abundance in fecal matter and high concentrations of butyrate were found to reduce anti-CTLA-4 immunotherapy efficacy in mouse models.^124^

The gut microbiome has similarly been implicated in anti-PD-1 immunotherapy. Ablation of the gut microbiome with antibiotics was shown to lower tumor growth in a mouse model of PDAC and with growth reduced further when combined with anti-PD-1 immunotherapy.^43^ Higher gut microbiome diversity has been associated with improved responses to anti-PD-1 immunotherapy.^108^ In particular, those that responded had more abundant bacteria belonging to order Clostridiales, family *Ruminococcaceae*, and genus *Faecalibacterium*, with these resulting in differences in immune recruitment.^108^ Patients with a higher abundance of *Faecalibacterium* spp. had better progression-free survival,^108^ similar to the response that has been shown with anti-CTLA-4 immunotherapy. Others have found that anti-PD-1 responders were enriched in *Akkermansia muciniphila*, the phylum Firmicutes, the family *Lachnospiraceae*, and the genera *Eubacterium* and *Enterococcus*^125^. Another group analyzing patient fecal samples showed that *Bifidobacterium longum*, *C. aerofaciens*, *Lactobacillus* spp., and *Enterococcus faecium* were among the bacterial species enriched in anti-PD-1 responders, though the authors do mention that this is likely an underestimate of the species based on their methods used^126^. FMT experiments in mice have previously shown that the addition of certain microbes can improve the activity of anti-PD-1 immunotherapy.^125^ A pair of clinical trials utilizing FMT in conjunction with anti-PD-1 immunotherapy in patients has shown success. In the first trial, donors had a higher abundance of *Lachnospiraceae* spp., *Veillonellaceae* spp., and *Ruminococcaceae* spp., with the post-treatment microbiota of responders including *Enterococcaceae* spp., *Enterococcus* spp., and *Streptococcus australis*.^127^ The second trial had donors with a higher abundance of the families *Lachnospiraceae*, *Ruminococcaceae*, *Bifidobacteriaceae*, and *Coriobacteriaceae*, with responders having higher amounts of cytotoxic T cells and changes in levels of circulating cytokines and chemokines^128^. SCFAs have also been implicated in anti-PD-1 efficacy, with patients with higher concentrations of acetic acid, propionic acid, butyric acid, and valeric acid having longer progression-free survival.^129^ The reasons why previous studies have shown that SCFAs are associated with different outcomes in anti-PD-1 immunotherapies and anti-CTLA-4 immunotherapies remain to be understood, but could be due to the different T cells that express these receptors or that SCFAs have different effects based on the SCFA and cancer type, as we have previously reviewed.^130^

As PD-1 and PD-L1 are associated as a receptor-ligand pair, with T cells expressing PD-1 and tumor cells expressing PD-L1, anti-PD-L1 immunotherapy has also been shown to be similarly dependent on the gut microbiome. Patients treated with antibiotics previously, but not concurrently, to anti-PD-1 or anti-PD-L1 immunotherapy have been shown to have worse overall survival across multiple types of cancer, including NSCLC and melanoma,^131^ further showing that the gut microbiota is implicated in both of these immunotherapies. Fecal microbiota analysis of a melanoma mouse model showed that the genus *Bifidobacterium* was associated with better anti-PD-L1 response^109^. Administration of *Bifidobacterium* spp. led to slower tumor growth and improved immune recruitment for multiple cancer types.^109^ In mice that have been colonized by only *Enterococcus* spp. and given anti-PD-L1 immunotherapy, *E. faecium* and *E. hirae* were among the species were associated with smaller tumors.^132^ In analyzing of *Enterococcus* spp., researchers found these species expressed a peptidoglycan hydrolase, SagA, which promoted anti-PD-L1 immunotherapy response, suggesting that disaccharide administration alongside anti-PD-L1 immunotherapy could have therapeutic potential.^132^

Researchers have also used a combination of 11 rare microbial species to support the anti-cancer effect of anti-CTLA-4 and anti-PD-1 immunotherapies in mouse models of adenocarcinoma and melanoma, leading to a decrease in tumor volume.^133^ In mice monocolonized with different species of bacteria, those that were colonized with *Bifidobacterium pseudolongum*, *L. johnsonii*, or *Olsenella* spp. had enhanced responses to immunotherapies across multiple models of cancer.^134^ Of these, *B. pseudolongum* was found to produce the metabolite inosine, which could improve anti-tumor immune functions.^134^ Additionally, the fungal order Capnodiales and its genus *Cladosporium*, commonly found as members of the human mycobiome, were shown to be more prevalent in melanoma patients that did not respond to immune checkpoint immunotherapy.^99^ The combination of anti-CTLA-4 and anti-PD-1 immunotherapies alongside supplementation with live *Clostridium butyricum* was shown to improve progression-free survival and response rate compared to the immunotherapies alone in patients with metastatic renal cancer in a recent phase I clinical trial^135^. While there are some bacterial species that in common between the immunotherapies mentioned, the lack of consensus between these papers, which could be attributed to the different patient populations studied or the different methods used, supports the need for studies focusing on larger patient populations from multiple locations and with multiple cancer types or meta-analyses of these published data sets.

The microbiome has also been implicated in other forms of immunotherapy, such as CpG-oligonucleotide immunotherapy, which has been used experimentally, but has not yet been FDA-approved. CpG oligodeoxynucleotides stimulate the immune system, due to their similarity bacterial DNA sequences, resulting in an inflammatory cascade that reduces tumor growth and causes necrosis. CpG-oligonucleotide immunotherapy efficacy was reduced in a colon cancer mouse model after administration of antibiotics, coupled with a lower rate of necrosis.^110^ Antibiotic treatment impaired tumor necrosis factor expression, as well as other pro-inflammatory responses, but the presence of some gut microbes reduced this effect, suggesting that the commensal gut microbiome improves the response of CpG-oligonucleotide immunotherapy.^110^ More specifically, the higher abundance of genera *Alistipes* and *Ruminococcus*, as well as the lower abundance of genus *Lactobacillus*, were found to be associated with better immune responses following CpG-oligonucleotide immunotherapy.^110^

As the FDA-approved targeted CAR-T therapies continue to increase in number, further studies on why patients may or may not respond have implicated the microbiome. An analysis of human patient populations receiving CAR-T therapy showed that patients receiving broad-spectrum antibiotics were more likely to have adverse outcomes and worse survival outcomes.^111^ Bacterial genera *Bacteroides*, *Ruminococcus*, *Eubacterium*, and *Akkermansia* were found to be most correlated with CAR-T response,^111^ with many of these also being implicated in immune checkpoint immunotherapy. SCFAs butyrate and pentanoate produced by gut bacteria *Megasphaera massiliensis* and *Megasphaera elsdenii* were shown to improve the abundance and activity of cytotoxic T cells.^136^ Further, when combined with murine CAR-T cells in a pancreatic mouse model, these metabolites were found to improve CAR-T immune responses, and similar improvements were seen with human CAR-T cells when exposed to these metabolites.^136^

### Allogenic hematopoietic transplants and graft-versus-host disease (GvHD)

Hematopoietic stem cell transplants are the transplant of stem cells derived from bone marrow or peripheral blood as a treatment for many blood cancers, including leukemia, lymphoma, and multiple myeloma, among other conditions. These stem cells can be derived from the patient from the patient (autologous) or, more commonly, from a donor (allogeneic). One of the common complications of allogeneic hematopoietic stem cell transplant (allo-HSCT) is GvHD, which occurs when the donor’s white blood cells from the graft attack the recipient. This condition can be life-threatening, but can also benefit patients, as the donor’s white blood cells can also attack tumor cells, as part of the graft-versus-tumor effect.

A higher abundance of *Eubacterium limosum* and other members of family *Eubacteriaceae* was found to be associated with a lower risk of cancer relapse and progression of disease following allo-HSCT.^112^ Reduced gut diversity has been associated with the occurrence of GvHD. ^137; 138^ and with a higher risk of death following allo-HSCT.^138^ Low levels of microbial tryptophan derivative 3-indoxyl sulfate in urine was found to be correlated with higher transplant-related mortality, with families *Lachnospiraceae* and *Ruminococcaceae* associated with increased 3-indoxyl sulfate and class Bacilli associated with decreased 3-indoxyl sulfate.^139^ Severe GvHD was associated with lower levels of the families *Lachnospiraceae*, including *Blautia* spp., and *Ruminococcaceae*.^113^ SCFAs are significantly lower in GvHD patients^113^ and have been shown to be protective against chronic GvHD.^140^ Others have shown that an increased abundance of genera *Blautia* was also associated with reduced GvHD lethality and improved survival.^141^ Similarly, genera *Enterococcus* was also associated with a higher incidence of GvHD, with lactose facilitating its growth.^142^ Gut metabolite trimethylamine N-oxide has also been associated with an increased risk of GvHD, due to increased immune activation.^143^ In a mouse model of GvHD, disease was associated with increased order Lactobacillales and deceased order Clostridiales, which was also noted in GvHD patients.^137^ Other studies utilizing mouse models have shown that increases in *Bacteroides* spp. are associated with reduced rates of GvHD, with the administration of *B. fragilis* reducing the development of GvHD.^144^

Building on this research, interventional studies focused on prebiotics and FMT have sought to exploit the proposed interaction between the gut microbiome and GvHD. Resistant starch and a nutritional supplement containing glutamine, fiber, and oligosaccharide were tested in patients undergoing allo-HSCT, with the prebiotics significantly lowering the incidence of GvHD, maintaining microbial diversity, and improving the prevalence of protective butyrate-producing bacteria.^145^ Another phase I clinical trial tested a fructo-oligosaccharide prebiotic known to increase SCFA levels in patients undergoing allo-HSCT and while the prebiotic was well-tolerated, the initial changes seen in the gut following ingestion were not sustained over time.^146^ A phase I/II clinical trial using FMT to treat patients with GvHD, found that patients receiving FMT had better clinical remission and overall survival, with microbiome analyses showing that diversity increased, abundance of the phyla *Proteobacteria* and *Firmicutes* decreased, and abundance of phylum *Bacteroidetes* increased.^147^ Another clinical trial using FMT to treat GvHD found that clinical response to FMT occurred in most patients, with some also being tapered off steroidal therapy, as FMT lead to increases in gut diversity and butyrate-producing bacteria, such as the family Clostridiales and *Blautia* spp.^148^ Unlike other therapies, there seems to be more of a consensus on the specific microbes and metabolites implicated in allo-HSCT and GvHD. While clinical trials have shown some success in manipulating the gut microbiome to prevent GvHD and improve patient outcomes, larger clinical studies with patients from multiple centers and locations are necessary to further show efficacy of these and if a combination of these interventions could be utilized in clinical settings.

### Others

The MEK pathway is involved in regulating growth and the cell cycle, and thus, therapies targeting this pathway can limit tumor growth. There are currently four FDA-approved MEK inhibitors, three of which are used for melanoma treatment. Prebiotics mucin and inulin have been shown to inhibit tumor growth in melanoma and colon cancer mouse models, due to increased *Bifidobacterium* spp. and *Akkermansia muciniphila* in the gut microbiome.^149^ Further, insulin has been shown to enhance the efficacy of an MEK inhibitor and reduce MEK inhibitor resistance in melanoma mouse models^149^, which has previously been shown to be a concern with this treatment. It remains to be determined if there would be similar improvements in patients undergoing treatment MEK inhibitor treatment when given these prebiotics, as this study was restricted to mouse models.

While FMT has been used extensively in cancer research to modulate the gut microbiome and in combination with other therapies, it has also been proposed as an independent cancer therapeutic. Previous research has shown that the wild mouse gut microbiota was better at protecting laboratory mice from colitis-associated CRC, as the wild gut microbiota was significantly different^150^. The possible role of FMT in cancer management and to mediate treatment-associated complications has been previously reviewed and discussed^151^, though further clinical trials are necessary further show the validity of FMT alone as an cancer treatment.

## Outlook and perspectives

The pathophysiological functions of noninfectious human-associated microbes have long been ignored. For this reason, the microbiome has only been implicated in cancer research over the past two decades, despite our long-established understanding of cancer and many therapeutic strategies. In the past few years, we have begun to understand that microbes in different tissue types are not merely contaminants and that many tissue types outside the gut have their own distinct microbiome, which can be disrupted in diseased states. Despite only a few microbes being categorized as those that can directly cause cancer, the number of microbes that indirectly affect cancer have steadily increased. Many recent studies have shown that the microbiomes of local tissues, other regions, and tumors themselves, can all affect cancer and its clinical care. As we have mentioned throughout this paper, there is a salient need for more longitudinal studies across large patient populations that focus on the impact of the microbiome in various types of cancers, to further understand how generalizable the previously implicated microbes we have described truly are and the possible mechanisms by which these microbes may be impacting cancer.

In this review, we have summarized the impacts of the gut and local microbiomes on various facets of cancer care, including development and progression, diagnostics, and therapeutics. Progress has also been made recently by multiple groups utilizing synthetic biology to re-engineer commensal microbes to elicit specific immune responses for various diseases, including cancer.^152–154^ As advances in research methods and analytical models continue to improve our knowledge about the microbes around and within us, we expect that the number of microbes associated with cancer will surge, expanding from the current set mainly focused on easily culturable bacteria to include microbes that were previously elusive. Further, these newly implicated microbes can serve as new targets to improve the efficacy of diagnostic and treatment methods, giving us new avenues to prevent cancer from occurring and progressing in patients and allowing clinicians to understand how to better improve responses to treatments.

## Data Availability

All data, models, and code generated or used during the study appear in the submitted article.
